# Supplementary material for: Inertial imitation method of MMC with hybrid topology for VSC-HVDC
Source: PLoS One. 2022 Dec 9;17(12):e0277553. doi: 10.1371/journal.pone.0277553 (PMC9733882; doi:10.1371/journal.pone.0277553)
Supplement: Attachment — Submitted filename: Response to Reviewers.docx [file pone.0277553.s002.docx]

Response to Reviewers

Dear Editor,

Thank you for allowing a revised version of our manuscript, with an opportunity to address the reviewers’ comments.

Best regards,

Wu Jie et al.

**Reviewer #1:**

There is no question.

**Reviewer #2:**

**1. No numerical results have been added in the abstract. The authors just mentioned "The results show...."**

The results were added in abstract as “The simulation results show that it can effectively improve the current control capability during the transient process for systems with a 1:2 ratio of converter capacity to grid capacity (The grid short-circuit capacity is 60MW and the MMC is 30 MW).” and “The results show that the power adjustment time of MMC under the proposed VSG control is about 1s, while the adjustment time under the conventional control strategy is greater than 4s.”

**2. "For VSG control structure and inertial equivalent method, there is still room for optimization." Is there any optimization the proposed method?**

The optimizable aspects are described separately from two aspects, which are modified and supplemented in the section 1 as follows:

For VSG control structure and inertial equivalent method, there is still room for optimization. For control structures, the modulation signal is directly output by the traditional VSG controller, without direct control of the AC current, and the fault ride-through capability is poor. Therefore, a suitable current inner loop can be constructed to improve the transient characteristics of the system. For inertial equivalent, the inertial energy in the aforementioned VSG method needs to be provided by an AC power grid, an energy storage system or a wind turbine, which requires an additional device or system to provide or consume the inertial energy. The power module capacitor of the MMC itself can be used to store energy and provide inertia.
